# Cardiopulmonary (No Ventilation) and Anesthetic Effects of Dexmedetomidine–Tiletamine in Dogs

**DOI:** 10.3389/fvets.2021.674862

**Published:** 2021-07-16

**Authors:** Zhiheng Zhang, Xueman Du, Hui Bai, Meilun Shen, Xiangying Ma, Rouqian Li, Xiaodi Jin, Li Gao

**Affiliations:** ^1^Heilongjiang Key Laboratory for Laboratory Animals and Comparative Medicine, College of Veterinary Medicine, Northeast Agriculture University, Harbin, China; ^2^College of Veterinary Medicine, Northeast Agricultural University, Harbin, China

**Keywords:** dexmedetomidine, tiletamine, dogs, intramuscular anesthesia, orchiectomy

## Abstract

The aim of the present study was to evaluate the anesthetic and cardiopulmonary effects of dexmedetomidine in combination with tiletamine (without zolazepam) as a general anesthetic. The study was divided into two phases. In Phase 1, 18 adult healthy mixed-breed dogs were randomly allocated into three groups: Group TD8 (4.5 mg kg^−1^ tiletamine and 8 μg kg^−1^ dexmedetomidine), Group TD10 (4.5 mg kg^−1^ tiletamine and 10 μg kg^−1^ dexmedetomidine), or Group TD12 (4.5 mg kg^−1^ tiletamine and 12 μg kg^−1^ dexmedetomidine). After drug administration, the heart rate (HR), respiratory rate (*f*_R_), mean arterial pressure (MAP), systolic arterial pressure (SAP), diastolic arterial pressure (DAP), peripheral hemoglobin oxygen saturation (SpO_2_), behavioral score, quality of induction and recovery, extent of ataxia, the time taken for induction, and the duration of anesthesia were recorded. The recovery time and quality were recorded after administration of atipamezole (50 μg kg^−1^) after 60 min. In phase 2, the feasibility of combining dexmedetomidine (10 μg kg^−1^) and tiletamine (4.5 mg kg^−1^) as general anesthetics for orchiectomy was evaluated in dogs (*n* = 6). HR, *f*_R_, MAP, SAP, DAP, temperature, SpO_2_, behavioral scores, and adverse reactions were recorded during each surgical procedure. In phase 1, the dogs were anesthetized for 5 min after administration of drugs and achieved a maximum behavioral score in TD10 and TD12 after 10 min. Although HR, MAP, SAP, DAP, and NIBP decreased in all three groups, they still maintained within the normal range. In phase 2, orchiectomy was completed smoothly in all dogs with little fluctuation in the physiological variables. We found that a combination of tiletamine (4.5 mg kg^−1^) and dexmedetomidine (10 μg kg^−1^) intramuscularly induced moderate anesthesia in dogs and could be utilized for short-term anesthesia and minor surgery.

## Introduction

Dexmedetomidine is a highly selective α_2_ adrenergic agonist that acts as a sedative and analgesic, with anti-anxiety properties. It displays a biphasic hemodynamic response. In particular, a low dose of dexmedetomidine can cause bradycardia and hypotension, while high doses can result in hypertension and exacerbate bradycardia (1–3).

Tiletamine is a competitive N-methyl-D-aspartic acid (NMDA) receptor antagonist that exhibits a dissociative anesthetic effect (4). It is highly species-specific and can induce analgesia and is a general anesthetic in mice, rats, pigeons, cats, and monkeys. However, tiletamine can cause depression in guinea pigs and rabbits (5, 6). It is usually provided with zolazepam in veterinary settings as a narcotic sedative (tiletamine/zolazepam or Zoletil^®^) for use as a short-term anesthetic for surgical procedures in dogs (7–9). It has been observed that dexmedetomidine–butorphanol–tiletamine–zolazepam (DBTZ) administered intramuscularly (IM) in healthy dogs rapidly induced short-term anesthesia and analgesia (7).

There are no published manuscripts of veterinary medicine in which dexmedetomidine and tiletamine alone have been used without zolazepam for anesthesia in dogs. We hypothesized that the combination of dexmedetomidine and tiletamine would provide stable short-term anesthesia with few side effects that could be used for minor surgical procedures. Thus, this combination was evaluated in order to eliminate this gap in the knowledge base in this field. Therefore, the present study was divided into two phases. In phase 1, we aimed to determine the efficacy of dexmedetomidine in combination with tiletamine in dogs using physiological parameters and evaluate the level of anesthesia. In phase 2, we evaluated the feasibility of combining dexmedetomidine and tiletamine as a general anesthetic for orchiectomy in dogs.

## Materials and Methods

### Animals

A total of 24 adult healthy mixed-breed dogs with American Society of Anesthesiologists physical status I (ASA I), weighing 5.6 ± 1 kg, and aged 1.5–3.1 years (12 males and 12 females) were used in the present study. The dogs were placed individually in clean and comfortable kennels and provided *ad libitum* access to a standard feed and water. All dogs were subjected to a basic physical examination, as described previously (10), and a complete blood count was performed to ensure the dogs were healthy. Exclusion criteria included irregular heart rhythm, body condition score >7 or <3 on a scale of 1 to 9, anemia (hematocrit < 25%), clinical signs of systemic disease, or additional drug administration within 48 h. The experiments were conducted in accordance with the guidelines of the Northeast Agricultural University Institutional Animal Care and Use Committee, Harbin, China. A total of 18 dogs were allocated into phase 1 while six male dogs were studied in phase 2. All dogs were adopted after experimentation.

### Phase 1: Cardiopulmonary and Anesthetic Effects of Tiletamine in Combination With Dexmedetomidine

#### Experimental Design and Grouping

All animals fasted for 12 h prior to anesthesia, but *ad libitum* access to water was permitted. Eighteen dogs were allocated into three equal groups (*n* = 6), using a random number table, namely, the TD8 group: 4.5 mg kg^−1^ tiletamine (Yuancheng Pharmaceutical Co. Ltd., Hubei, China) and 8 μg kg^−1^ dexmedetomidine (Zoties Ltd., NY, USA), the TD10 group: 4.5 mg kg^−1^ tiletamine and 10 μg kg^−1^ dexmedetomidine, and the TD12 group: 4.5 mg kg^−1^ tiletamine and 12 μg kg^−1^ dexmedetomidine. Powdered tiletamine (250 mg) was dissolved in 5 ml of normal saline for a final concentration of 50 mg/ml. Each combination of drugs was mixed within the same 2.5-ml syringe and administered as an IM injection into the quadriceps muscle. When no response to mechanical stimulation was observed, including the sound of a handclap close to the dog's ears or a tail clamp using Kocher's forceps for 3 s, each dog was placed in a right lateral recumbent position, with the tongue placed outside of the mouth to allow unrestricted breathing.

#### Assessment of Physiological Values

Heart rate (HR), respiratory rate (*f*_R_), mean arterial pressure (MAP), systolic arterial pressure (SAP), diastolic arterial pressure (DAP), peripheral hemoglobin oxygen saturation (SpO_2_), and temperature (T) were recorded after 0, 5, 10, 20, 30, 40, 50, and 60 min using a multiple physiological parameter monitor (iMCE8 Vet, Mindray, Shenzhen, China). SPO_2_, T, and HR were measured using an SpO_2_ tongue probe, temperature probe (in the esophagus), and electrocardiograph (ECG) clips (on the skin). Non-invasive blood pressure (NIBP, including MAP, SAP, and DAP) was measured by oscillometry using a blood pressure cuff (3^#^, 5–13 cm) on the right thoracic limb, slightly distal to the elbow. HR and *f*_*R*_ were measured manually prior to induction of anesthesia using the multiple physiological parameter monitor, or manually following induction.

#### Quality Assessment of Anesthesia

Three evaluators blinded to the experimental details independently assessed the quality of induction and recovery, the degree of ataxia, and behavioral scores. The mean scores were recorded. The quality of induction and recovery and the degree of ataxia were evaluated using a standardized scoring system (11): 0 represents an animal that can walk without ataxia with smooth induction/recovery; 1 indicates an animal that can walk with minimal ataxia with uncomplicated induction/recovery; 2 indicates mild ataxia with difficult induction/recovery; and 3 represents a dog that walks with significant ataxia/crawling with rough induction/recovery. The quality of recovery was scored using a three-point scale: (1) smooth, quiet, non-vocal without paddling or flailing; (2) some vocalization, paddling, or uncoordinated movement but of short duration and easily calmed; and (3) emergence delirium with excessive vocalizing and uncoordinated movement. In addition, the time required for induction of anesthesia, its duration, and the length of recovery were recorded. Recovery time (RT) was recorded after atipamezole (50 μg kg^−1^) was injected after 60 min. For dogs with poor recovery score, 2 μg kg^−1^ dexmedetomidine was administered to improve the quality of recovery. Behavioral scores were measured using methods previously reported (12, 13) after 5, 10, 20, 30, 40, 50, and 60 min. The total score was the sum of posture, sedation, analgesia, skeletal muscle relaxation, and auditory response score (Table 1). The greater the score, the better the quality of anesthesia. A maximum score of 16 indicated excellent anesthesia, while scores of 11–15 indicated anesthesia that was moderate, and a score <11 indicated mild anesthesia (12, 13).

**Table 1:** Behavioral score.

**Criteria**	**Score**	**Observation**
Posture	0	Standing
	1	Sitting or ataxic, but able to walk
	2	Completely prone, unable to walk
	3	Sternal recumbency, but able to move the tail or paw
	4	Lateral recumbency without movement
Sedation	0	Normal
	1	Mild sedation (recumbent, head down, strong palpebral reflex, normal eye position)
	2	Moderate sedation (recumbent, head down, moderate palpebral reflex, partial ventromedial eye rotation)
	3	Profound sedation (recumbent, head down, palpebral reflex absent, complete ventromedial eye rotation)
Nociception^*^	0	Normal response
	1	Reduced response
	2	Reduced response
	3	No response
Jaw relaxation	0	Normal resistance to opening the mouth
	1	The jaw can be opened, but there is still some resistance
	2	Little resistance to opening the mouth and obvious muscle relaxation
	3	No response
Auditory response^†^	0	Normal response
	1	Mild decrease in response (eye movement with body movement)
	2	Moderate decrease in response (eye movement without body movement)
	3	Profound decrease in response (no movement)

* *Reflex withdrawal to clamping of the tail, the skin of body surface at the paramedian abdomen, and the interdigital web of the foot of all limbs for 3 s using Kocher's forceps*.

† *Response to noise created by a handclap close to the animal's ears*.

#### Adverse Events

The occurrence of adverse events was recorded, including myoclonus, regurgitation, cardiac arrest, hypoventilation (*f*_R_ < 6 breaths min^−1^), hypoxemia (SpO_2_ < 90%), bradycardia (HR < 60 beats min^−1^), hypotension (MAP < 60 mmHg), or hypothermia (T < 36.6°C). During the experiment, if adverse events were observed, the dog was excluded from the study and treated. Myoclonic activity can be decreased with intravenous administration of midazolam. Once regurgitation occurred, treatment should be symptomatic, to include antibiotics, oxygen, and the prevention of aspiration pneumonia. Cardiopulmonary resuscitation (CPR) and epinephrine were used in the treatment of cardiac arrest. Dogs showing signs of hypoventilation or hypoxemia were intubated, manually ventilated, and supplemented with 100% oxygen. When bradycardia, hypotension, and hypothermia presented, repeated administration of atropine, IV fluids, and heating pad was indicated for treatment.

### Phase 2: Tiletamine in Combination With Dexmedetomidine Used in a Surgical Procedure

#### Preoperative Preparation and Surgical Procedure

Six male dogs underwent orchiectomy in the phase 2 study. The dogs fasted overnight with *ad libitum* access to water. Doses of tiletamine and dexmedetomidine in phase 2 were selected on the basis of an assessment of quality of anesthesia of the three groupings in Phase 1. A 22-G peripheral intravenous catheter (Hongda Co. Ltd., Jiangxi, China) was placed aseptically into a cephalic vein, and lactated Ringer's solution (Hengfengqiang Biotechnology Co. Ltd., Jiangsu, China) was administered as an intravenous (IV) fluid infusion using an infusion pump (WIT-601A, WIT Medical Technology Co., Ltd, China) at a rate of 5 ml kg^−1^ h^−1^. Dexmedetomidine (10 μg kg^−1^) and tiletamine (4.5 mg kg^−1^) were administered by IM injection into the quadriceps muscle. Once recumbent, each dog was placed on the operating table to prepare for surgery using 100% oxygen supplied by mask. After the surgical site was shaved then scrubbed, lidocaine (2 mg kg^−1^, Hualu Pharmaceutical Co. Ltd., Shandong, China) was injected into the testes prior to a cutaneous incision of the anterior scrotum was created. Six dogs underwent open orchiectomy (14) using a prescrotal approach with the dogs in a lateral recumbent position and the upper pelvic limb elevated in a flexed position. The pampiniform plexus and vas deferens were ligated together using a transfixion ligature of 3–0 polyglycolic acid (Jinhuan Medical Products Co., Ltd., Shanghai, China). The subcutaneous tissue and skin were closed with a single suture using a simple interrupted pattern. All surgical procedures were performed by the same veterinary student. All dogs were administered amoxicillin–clavulanate potassium (15 mg kg^−1^ IM; Zoetis, Borgo San Michele, Latina, Italy) and meloxicam (0.2 mg kg^−1^ IM; Boehringer Ingelheim, Barcelona, Spainy) following surgery.

#### Measurement of Parameters

HR, *f*_R_, MAP, SAP, DAP, T, SpO_2_, and behavioral scores were recorded 0, 5, 10, 20, 30, 40, 50, and 60 min after administration of dexmedetomidine and tiletamine. The start time of surgery, the duration of surgery (min) from incision to completion of suturing, and the time from placing the final skin suture until the dog was standing were recorded. Adverse reactions were recorded (myoclonus, regurgitation, cardiac arrest, hypoventilation, hypoxemia, bradycardia, hypotension, or hypothermia) during the entire procedure.

### Statistical Analysis

Sample sizes were calculated on the basis of detecting a difference of 10% in behavioral score between the different groups of drug concentrations, with a standard deviation (SD) of 2, achieving an 80% statistical power and an overall alpha level of 0.05. Thus, a minimum of six dogs per group was required.

All statistical analyses were performed using SPSS Statistics v25 software (IBM, NY, USA). The normality of the distribution of data was assessed using a Shapiro–Wilk test. The data are expressed as means ± standard deviation (parametric variables) or medians (min–max) (non-parametric variables), as appropriate. Depending on the distribution, a one-way ANOVA followed by Tukey's *post hoc* analysis was used to assess differences between groups in terms of induction time, duration, and recovery. A Kruskal–Wallis test and Dunn's multiple-comparison test were used to assess differences between groups in terms of induction quality score, recovery quality score, and ataxia score. Normally distributed physiological parameters (HR, *f*_R_, MAP, SAP, DAP, T, and SpO_2_) were analyzed using a general linear mixed model analysis (time, treatment, and their interactions are fixed effects, and individual dogs are random effects). *Post-hoc* multiple comparisons were performed using Dunnett's test to compare each time point with the baseline (0 min) and using Tukey's test to compare different treatment groups simultaneously. Behavioral scores were analyzed using a generalized linear mixed-effect model assuming a Poisson family with a logarithmic link function, while a Kruskal–Wallis test was used to compare scores between treatment groups and the time point with the baseline (0 min). For all analyses, *p* < 0.05 was considered significantly different.

## Results

All 18 dogs completed the study and recovered from anesthesia in phase 1. Table 2 displays the quality of induction and recovery, the degree of ataxia, the time required for induction of anesthesia, its duration, and time for recovery. The dogs appeared to exhibit rapid induction to a recumbent position with a slow, calm recovery in the TD8, TD10, and TD12 groups. Two dogs in TD8 and one in TD10 experienced delirium during recovery. There were significant differences in induction time, duration time, and recovery time among the three groups (*p* = 0.003, 0.001, and 0.020, respectively). As the concentration of dexmedetomidine increased, the induction time gradually shortened, and the duration and recovery time gradually increased in the TD8, TD10, and TD12 groups. The dogs in each group were anesthetized for 5 min after administration of the drugs. The score for induction quality, recovery quality, and ataxia showed no significant difference between TD8, TD10, and TD12 (*p* > 0.05).

**Table 2:** The quality of induction and recovery, the degree of ataxia, and the time of induction, duration, and recovery in dogs that were anesthetized with tiletamine and three concentrations of dexmedetomidine in phase 1.

**Variables**	**Groups**		
	**TD8**	**TD10**	**TD12**
Time of induction (min)	3.8 ± 0.8	2.8 ± 0.5^*^	2.2 ± 0.7^*^
Time of duration (min)	37.3 ± 9.2	47.7 ± 11.3	54.0 ± 6.6^*^
Time of recovery (min)	4.1 ± 0.5	6.2 ± 2.5^*^	7.9 ± 2.9^*^^#^
Induction quality (scale: 0–3)	1.5 (0–2)	1.0 (0–2)	0.5 (0–1)
Recovery quality (scale: 0–3)	1.0 (0–1)	1.0 (0–2)	1.0 (0–2)
Ataxia (scale: 0–3)	1.5 (1–2)	1.5 (1–3)	1.5 (0–3)

*TD8 group (n = 6), 4.5 mg kg^−1^ tiletamine plus 8 μg kg^−1^ dexmedetomidine; TD10 group (n = 6), 4.5 mg kg^−1^ tiletamine plus 10 μg kg^−1^ dexmedetomidine; TD12 group (n = 6), 4.5 mg kg^−1^ tiletamine plus 12 μg kg^−1^ dexmedetomidine*.

*Mean ± standard deviation is displayed for normally distributed outcome variables while the median (min–max) was included as the measure of central tendency for non-normal data*.

* *Significantly different from TD8 (P < 0.05)*.

# *Significantly different from TD10 (P <0.05)*.

**Figure 1:**
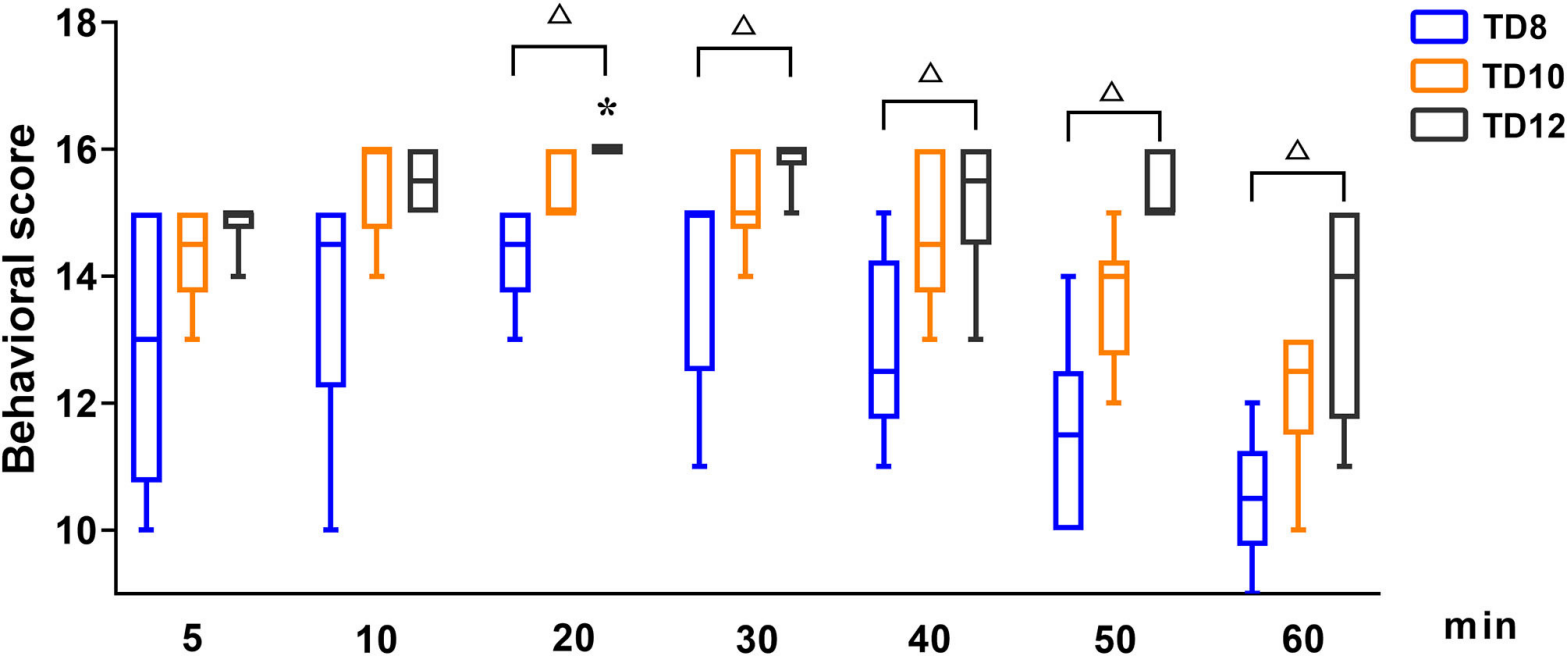
The behavioral scores in dogs undergoing anesthesia with tiletamine and three concentrations of dexmedetomidine at Phase 1. TD8 group (*n* = 6), 4.5 mg kg^−1^ tiletamine plus 8 μg kg^−1^ dexmedetomidine; TD10 group (*n* = 6), 4.5 mg kg^−1^ tiletamine plus 10 μg kg^−1^ dexmedetomidine; TD12 group (*n* = 6), 4.5 mg kg^−1^ tiletamine plus 12 μg kg^−1^ dexmedetomidine. Values are expressed as medians (min–max). *Significantly different from 5 min at the same group (*p* < 0.05). ^#^Significantly different from TD10 within the same time (*p* < 0.05). ^Δ^ Significantly different from TD12 within the same time (*p* < 0.05).

The behavioral score at phase 1, shown in Figure 1, was statistically significant among treatment groups (*p* = 0.016), time (*p* = 0.001), and their interaction (*p* = 0.004). For each treatment group in phase 1, the behavioral score was significantly lower in TD8 than that in TD10 (*p* < 0.001) and TD12 (*p* < 0.001), while the score in TD12 was obviously higher than that in TD10 (*p* = 0.002). The overall trend in behavioral score was increased firstly and then decreased for all treatment groups. For the interaction of treatment groups and time, the TD12 group was obviously higher than the TD 8 group (*p* = 0.005).

**Figure 2:**
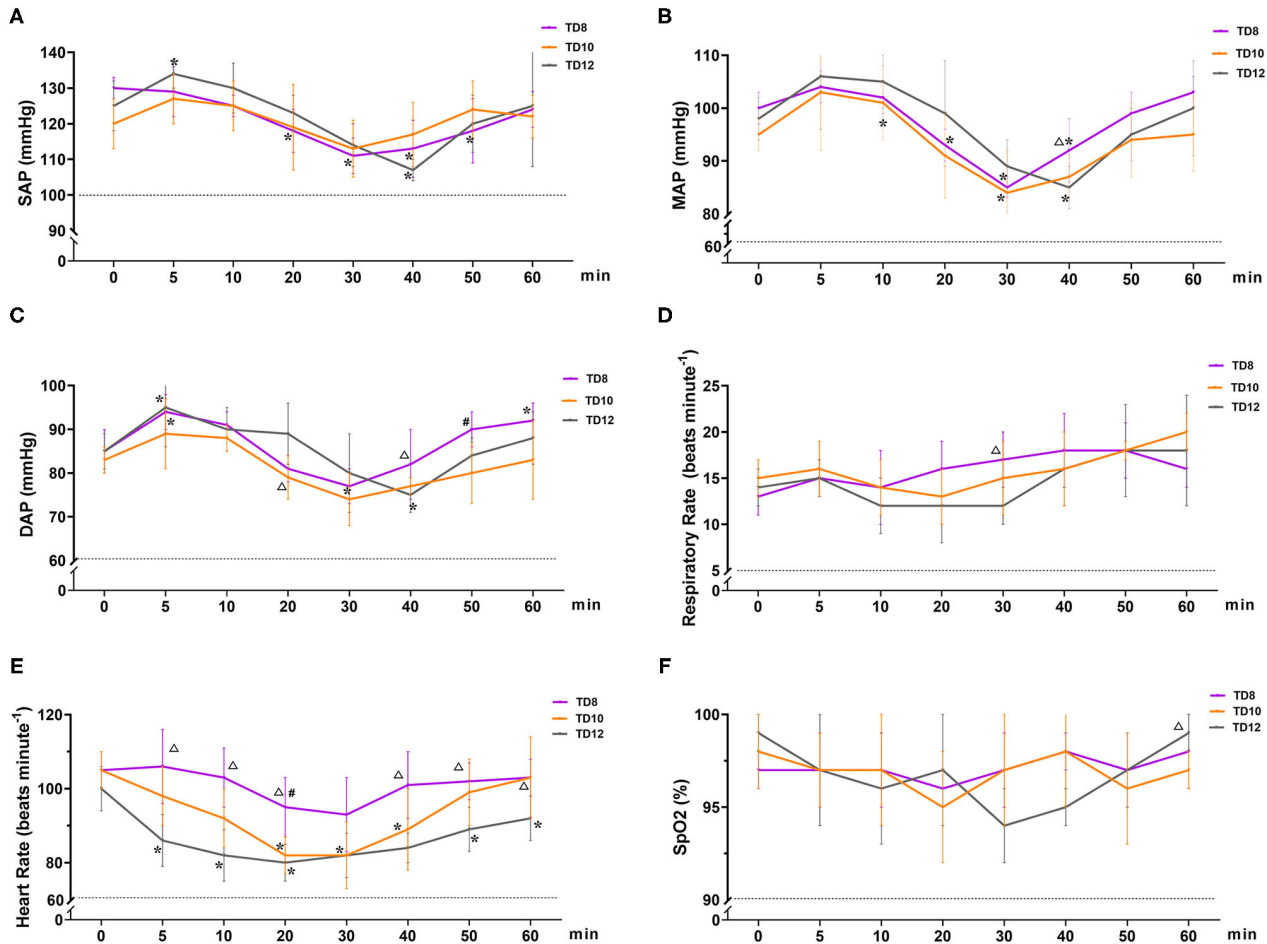
Cardiopulmonary variables in dogs that were administered tiletamine and three concentrations of dexmedetomidine in Phase 1. **(A)** Systolic arterial pressure (SAP); **(B)** mean arterial pressure (MAP); **(C)** diastolic arterial pressure (DAP); **(D)** respiratory rate (*f*_R_); **(E)** heart rate (HR); and **(F)** peripheral hemoglobin oxygen saturation (SpO_2_). TD8 group, 4.5 mg kg^−1^ tiletamine plus 8 μg kg^−1^ dexmedetomidine; TD10 group, 4.5 mg kg^−1^ tiletamine plus 10 μg kg^−1^ dexmedetomidine; TD12 group, 4.5 mg kg^−1^ tiletamine plus 12 μg kg^−1^ dexmedetomidine. Values are expressed as mean ± standard deviation (SD). *Significantly different from 0 min at the same group (*p* < 0.05). ^#^Significantly different from TD10 within the same time (*p* < 0.05). ^Δ^ Significantly different from TD12 within the same time (*p* < 0.05).

The physiological parameter variables in phase 1 are shown in Figure 2. The overall trend in the arterial blood pressure for all treatment groups was increasing (0~5 min), decreasing (5~30 or 40 min), and increasing (40~60 min), but without hypotension. SAP, DAP, and MAP [normal value: 100~160, 60~100, and 80~120 mmHg (15), respectively] varied significantly over time in the three groups (*p*_time_ = 0.001, 0.008, and 0.002, respectively). The interaction between treatment and time of SAP, DAP, and MAP was not statistically significant (*p* = 0.186, 0.174, and 0.530, respectively). There were no significant differences in SAP, DAP, and MAP among treatment groups (*p* = 0.201, 0.392, and 0.388, respectively).

HR [normal value (15): 80~160 beats min^−1^] decreased and then gradually recovered over time, and the mean HR declined from baseline by 10~20 beats per min. HR varied significantly in the treatment group (*p* = 0.001), but not significantly over time (*p*_time_ = 0.344) and their interactions (*p* = 0.913). In terms of HR for the three groups, TD8 was higher than TD12 after 5, 10, 20, 40, 50, and 60 min (*p* = 0.003, 0.001, 0.003, 0.012, 0.014, and 0.048, respectively). HR in TD10 was lower than TD8 at 20 minutes (*p* = 0.011).

During the phase 1 study, SpO_2_ [normal value (15): 90~100%), *f*_R_ [normal value (15): 10–25 beats minute^−1^], and temperature [normal value (15): 37.0 ~39.5°C] were not statistically different among time, treatment groups, and their interaction, and all the values were within the normal range. The temperature of all dogs in phase 1 was 38.7 ± 0.3°C. No hypoxia, hypotension, bradycardia, or bradypnea was observed in phase 1.

**Figure 3:**
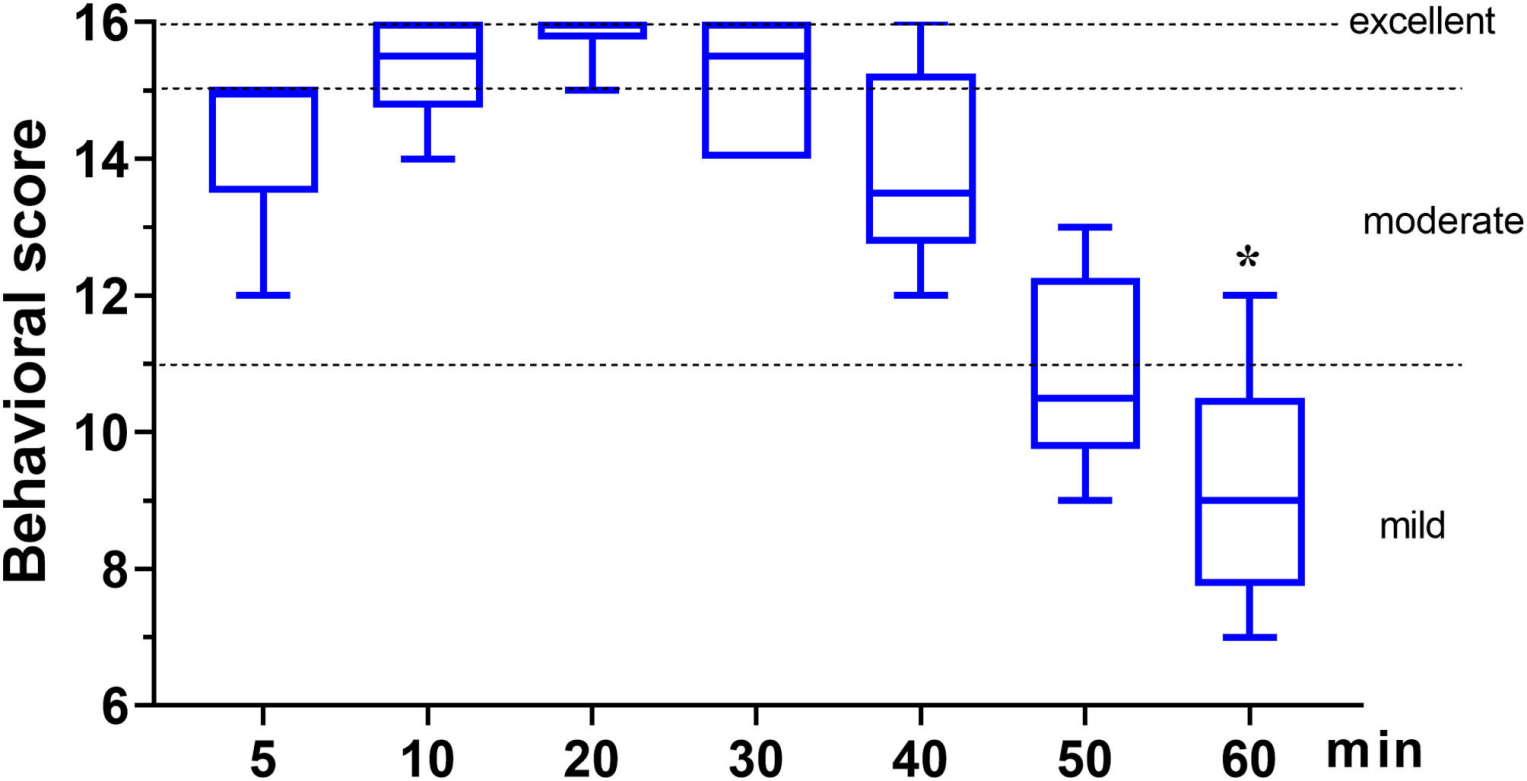
The behavioral score in dogs (*n* = 6) undergoing orchiectomy administered with 10 μg kg^−1^ dexmedetomidine and 4.5 mg kg^−1^ tiletamine intramuscularly at Phase 2. Values are expressed as medians (min–max). *Significantly different from 5 min (*p* < 0.05).

**Figure 4:**
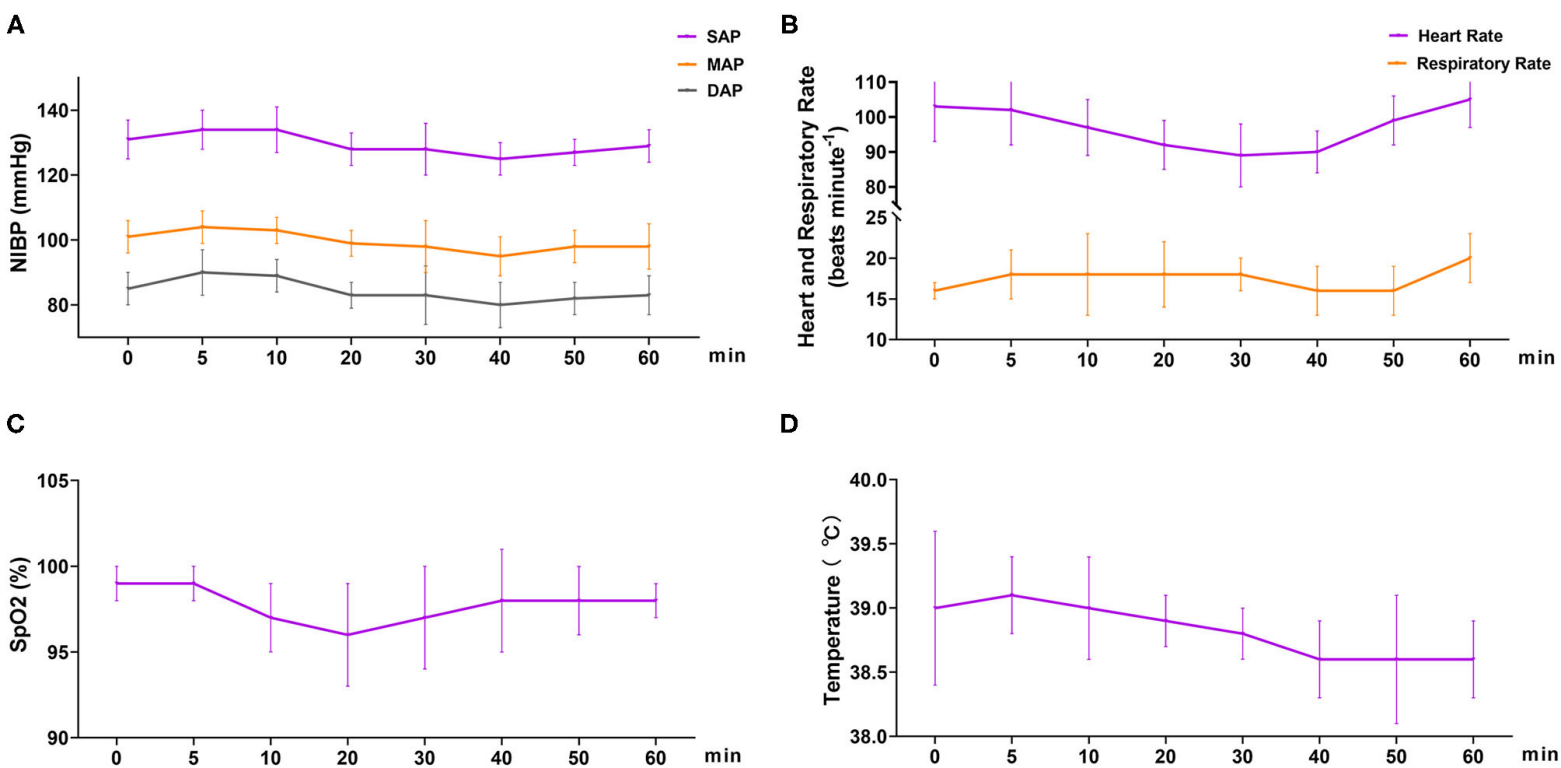
Cardiopulmonary variables in dogs (*n* = 6) undergoing orchiectomy administered with 10 μg kg^−1^ dexmedetomidine and 4.5 mg kg^−1^ tiletamine intramuscularly at Phase 2. **(A)** Systolic arterial pressure (SAP), mean arterial pressure (MAP), and diastolic arterial pressure (DAP); **(B)** heart rate (HR) and respiratory rate (*f*_R_); **(C)** peripheral hemoglobin oxygen saturation (SpO_2_) and **(D)** temperature (T). Values are expressed as mean ± standard deviation (SD). *Significantly different from 0 min at the same group (*p* < 0.05).

In phase 2, all six dogs underwent orchiectomy smoothly, with no apparent movement or pain during surgery. Behavioral score (Figure 3) changed significantly over time (*p* < 0.001), but was only significantly different from the baseline (5 min) 60 min after the start of surgery (*p* = 0.043). The mean time for a behavioral score >11 (in minutes, with 95% confidence interval) was 51 (95% CI = 43 to 59). Surgery start time and the duration time of surgery (starting time from incision to completion) in the six dogs was 19.8 ± 7.4 and 11.7 ± 4.3 min, respectively (Table 3). The physiological parameters (SAP, DAP, MAP, HR, T, and SpO_2_) did not vary significantly over time (*p*_time_ = 0.341, 0.250, 0.503, 0.290, 0.545, and 0.915, respectively), except for *f*_R_ (*p*_time_ = 0.030) (Figure 4). No hypoxia, hypotension, bradycardia, or bradypnea was observed during phase 2. Complications associated with surgery were not observed. The sutures were removed 10–14 days post surgery.

**Table 3:** Time related with surgical procedures at Phase 2.

**Variables**	**Times (minutes)**
Surgery start time	19.8 ± 7.4
Surgery time from incision to suture completion	11.7 ± 4.3
The time from placing the last skin suture until the dog standing	21.8 ± 9.4

*Values are expressed as mean ± standard deviation (SD)*.

## Discussion

In the study reported here, cardiorespiratory and anesthetic effects were evaluated after IM administration of tiletamine–dexmedetomidine (phase 1). Subsequently, the feasibility of combining dexmedetomidine (10 μg kg^−1^) and tiletamine (4.5 mg kg^−1^) as a general anesthetic in canine orchiectomy was evaluated (phase 2). In phase 1, rapid and smooth anesthetic induction was observed in three treatment groups (groups TD8, TD10, and TD12), with few adverse events (emergence delirium). Moderate anesthesia is defined as a behavioral total score > 11 (12, 13). The behavior score increased then gradually reduced over time in three treatment groups, and the score of TD8 was significantly decreased compared with TD10 and TD12. Our results showed that the TD10 and TD12 groups provided moderate anesthesia for 40~50 min in dogs.

As is well-known, tiletamine–zolazepam can result in long and difficult recoveries in some animals (16, 17). In the present study, dogs treated with tiletamine and dexmedetomidine showed fewer dissociation symptoms during the period of recovery. Studies have shown that tiletamine underwent hepatic metabolism and renal excretion (18, 19). In cats, tiletamine is metabolized more rapidly than zolazepam, resulting in a smoother recovery. However, for dogs, the reverse is true. The metabolism of tiletamine is longer than zolazepam, and so dissociative effects are observed, including muscle rigidity, sympathetic stimulation, and the emergence of delirium (20). Of the 18 dogs in phase 1, two in group TD8 and one in group TD10 experienced dissociative effects with the emergence delirium after administration of atipamezole (50 μg kg^−1^, IM). We speculate that the results in this study occurred for two reasons: (1) the metabolic rate of tiletamine is greater than that of dexmedetomidine, and (2) the sedative effect of dexmedetomidine is still partially retained after treatment with low-dose atipamezole. For dogs with poor recovery score, 2 μg kg^−1^ dexmedetomidine improved their recovery quality. These results also proved our conjecture.

Dexmedetomidine can cause cardiovascular side effects, such as transient blood pressure changes, HR decreases, cardiac output decreases, and systemic vascular resistance increases. Generally, it is used clinically in combination with other drugs, such as opioids, benzodiazepines, and phencyclidines to produce synergistic effects, which can significantly enhance their efficacy and reduce adverse events (21–24). Tiletamine, similar in chemical structure and pharmacology to ketamine, is a dissociative anesthetic that can antagonize NMDA receptors (25). In the study reported here, tiletamine and dexmedetomidine were used to the dog anesthesia, and the trend in changes of arterial blood pressure was similar in three treatment groups. SAP, DAP, and MAP were sight increasing (0~5 min), decreasing (5~30 or 40 min), and increasing (40~60 min) subsequently, without hypertension (MAP > 140 mmHg). A similar result was reported in dogs that received dexmedetomidine (26) or dexmedetomidine–butorphanol–tiletamine–zolazepam treatments (7). However, the increase in arterial blood pressure did not cause hypertension in the three treatment groups. Dexmedetomidine at 15 and 20 μg/kg increased arterial blood pressure in a stepwise fashion, and the hypertension caused by dexmedetomidine was related to the dose (27). We speculate that the temporary increase of MAP following administration was attributable to the lower dose (8–12 μg/kg) of dexmedetomidine included in this drug combination.

HR decreased then gradually recovered over time, mean HR declining in from baseline by 10~20 beats per minute. HR showed significant differences in three treatment groups, which indicated that the effect of dexmedetomidine in reducing HR is dose-dependent (28). Similarly, no bradycardia was observed in three treatment groups. The normal range of HR was believed to be that sympathetic stimulation of tiletamine alleviated the negative effects of dexmedetomidine on HR (7).

SpO_2_, *f*_R_, and temperature in the three treatment groups have no significant differences, and they were all within the normal range. Therefore, we believed that the combination of dexmedetomidine and tiletamine displayed relatively lesser influence on SpO_2_, *f*_R_, and temperature in dogs.

Orchiectomy is one of the most commonly performed surgeries in companion animal practice (29). In phase 2, the feasibility of combining dexmedetomidine and tiletamine as general anesthetics was evaluated in dogs. The dose of tiletamine and dexmedetomidine in phase 2 was chosen from an assessment of anesthesia quality among the three drug concentrations in phase 1. For orchiectomy, we expected the total duration of surgery to be ~30–40 min. Based on this, we selected 10 μg kg^−1^ dexmedetomidine and 4.5 mg kg^−1^ tiletamine for phase 2. As expected, our results in phase 2 revealed that the combination of tiletamine and dexmedetomidine can provide moderate anesthesia [51 min (95% CI = 43 to 59)] and complete the operation without complications.

There were a number of limitations in the present study. Although we monitored the basic physiological parameters (HR, *f*_R_, MAP, SAP, DAP, T, and SpO_2_) in the present study, ventilation (capnography or blood gas analysis), as an important physiological function, was not monitored due to the limitation in the available equipment. Additionally, tiletamine was not easy to obtain. Anesthesia was evaluated using the behavioral score, a system created using other species, in which the degree of sedation, analgesia, and muscle relaxation could be evaluated. We found that the threshold values for depth of anesthesia were similar in the silver fox and the dog. In the future, our studies will assess the detailed effectiveness of behavioral score for canine anesthesia.

In conclusion, we found that the combination of tiletamine (4.5 mg kg^−1^) and dexmedetomidine (10 μg kg^−1^) administered IM induced moderate anesthesia in dogs for ~40~50 min. Thus, we believe that this drug combination can be used for short-term anesthesia and orchiectomy in dogs.

## Data Availability Statement

The original contributions generated for the study are included in the article/supplementary material, further inquiries can be directed to the corresponding author/s.

## Ethics Statement

The animal study was reviewed and approved by Northeast Agricultural University Institutional Animal Care and Use Committee.

## Author Contributions

ZZ: study design, data collection, data analysis, writing of the first draft of the manuscript, review, and editing of manuscript. XD: study design, data management, experiments performed, review of manuscript. XM and HB: data collection, data analysis, review, and editing of manuscript. MS: data collection, statistical analysis, and review of manuscript. RL and XJ: statistical analysis, interpretation of results, and manuscript preparation. LG: conceived and designed the experiments and critical revision of manuscript. All authors read and approved the final manuscript.

## Conflict of Interest

The authors declare that the research was conducted in the absence of any commercial or financial relationships that could be construed as a potential conflict of interest.
